# Race-specific genotypes of *Pseudomonas syringae* pv. tomato are defined by the presence of mobile DNA elements within the genome

**DOI:** 10.3389/fpls.2023.1197706

**Published:** 2023-07-05

**Authors:** Benedetta Orfei, Joël F. Pothier, Linda Fenske, Jochen Blom, Chiaraluce Moretti, Roberto Buonaurio, Theo H. M. Smits

**Affiliations:** ^1^ Dipartimento di Scienze Agrarie, Alimentari e Ambientali (DSA3), Università degli Studi di Perugia, Perugia, Italy; ^2^ Environmental Genomics and Systems Biology Research Group, Institute of Natural Resource Sciences (IUNR), Zurich University of Applied Sciences ZHAW, Wädenswil, Switzerland; ^3^ Bioinformatics and Systems Biology, Justus-Liebig University Giessen, Giessen, Germany

**Keywords:** *Pseudomonas avellanae*, *Pseudomonas syringae* pv. tomato, comparative genomics, AvrPto1, AvrPtoB, race shift

## Abstract

*Pseudomonas syringae* pv. tomato is the causal agent of bacterial speck of tomato, an important disease that results in severe crop production losses worldwide. Currently, two races within phylogroup 01a (PG01a) are described for this pathogen. Race 0 strains have avirulence genes for the expression of type III system-associated effectors AvrPto1 and AvrPtoB, that are recognized and targeted by the effector-triggered immunity in tomato cultivars having the *pto* race-specific resistance gene. Race 1 strains instead lack the *avrPto1* and *avrPtoB* genes and are therefore capable to aggressively attack all tomato cultivars. Here, we have performed the complete genome sequencing and the analysis of *P. syringae* pv. tomato strain DAPP-PG 215, which was described as a race 0 strain in 1996. Our analysis revealed that its genome comprises a 6.2 Mb circular chromosome and two plasmids (107 kb and 81 kb). The results indicate that the strain is phylogenetically closely related to strains Max13, K40, T1 and NYS-T1, all known race 1 strains. The chromosome of DAPP-PG 215 encodes race 1-associated genes like *avrA* and *hopW1* and lacks race 0-associated genes like *hopN1*, giving it a race 1 genetic background. However, the genome harbors a complete ortholog of *avrPto1*, which allows the strain to display a race 0 phenotype. Comparative genomics with several PG01a genomes revealed that mobile DNA elements are rather involved in the evolution of the two different races.

## 1 Introduction

Bacterial speck is one of the most widespread and economically important disease of tomato (Schneider & Grogan, 1977). Its symptoms affect leaves, flowers, fruits and stems of the plant and consist in small, irregular dark brown to black necrotic spots (Goode & Sasser, 1980). Usually surrounded by a yellow halo (green in the fruit), these spots can converge in the leaves as they appear, increasing the necrotic area of the foliage, and thus leading to plant death. Yield losses for outbreaks of this disease could be around 20-25% for tomato seedlings and up to 75% for tomato fruits (Yunis et al., 1980; Basim et al., 2004). The causative agent of the disease is *Pseudomonas syringae* pv. tomato, a Gram-negative, rod-shaped bacteria ubiquitously widespread in all tomato crop growing areas. It is a member of the *P. syringae* species complex, one of the most relevant groups of phytopathogenic bacteria (Mansfield et al., 2012) with its 15 species assigned to 13 different phylogroups (PGs) (Berge et al., 2014). *P. syringae* pv. tomato falls into one of the two clades of the phylogroup 01 (PG01a), while in the other clade (PG01b), *Pseudomonas avellanae* and *P. syringae* pv. actinidiae are included.

In order to begin its cycle of infection and cause disease, *P. syringae* pv. tomato can occur as a consequence of natural phenomena like rain and wind, water irrigation, and also due to the use of contaminated seeds. As an epiphyte, *P. syringae* pv. tomato can survive and grow on the surface of the leaves and the seeds. Then, when the environmental conditions are favorable, it can rapidly increase its population and enter the host plant trough natural openings or wounds exploiting its polar flagella.

Inside the plant, the apoplast is the niche used by *P. syringae* pv. tomato for its colonization and subsequentially infection (Melotto et al., 2008). Pathogenicity and virulence of *P. syringae* pv. tomato largely rely on the use of the Hrp (hypersensitive response and pathogenicity) Type III Secretion System (T3SS) and its Type III Effectors (T3Es) like Hop (Hrp outer protein) or Avr (avirulence) proteins, as well as the production of the phytotoxin coronatine. In *P. syringae* pv. tomato DC3000, the genes *hopM1* and *avrE1* are both required for the establishment of an aqueous environment in the apoplast that promotes growth and thus colonization of the pathogen, while *avrPto1* and *avrPtoB* suppress the pattern-triggered immunity (PTI) of the plant, usually elicited by a structural component of the bacteria flagellum, flagellin, encoded by *fliC* (He et al., 2016).

The discovery and subsequent cloning in cultivated tomato strains of the *pto* gene, encoding a serine-threonine kinase capable of recognizing both the AvrPto1 and AvrPtoB protein effectors and activating the effector-triggered immunity (ETI) of the host (Xiao et al., 2007; Kunkeaw et al., 2010), allowed some control of the disease for a few decades. However, the emergence of *P. syringae* pv. tomato strains lacking functioning AvrPto1 and AvrPtoB effectively rendered the protection offered by Pto ineffective and allowed the establishment of a new race (race 1) of *P. syringae* pv. tomato capable of causing disease even in plants that carries the *pto* gene. Even today, the capability to cause disease in resistant tomato plants is the basis of the race assignment process for *P. syringae* pv. tomato, although there are useful genotypic markers to differentiate between the two different races (Jones et al., 2015). Moreover, strains of *P. syringae* pv. tomato expressing an intermediate phenotype between race 0 and race 1 strains have been characterized as well (Kraus et al., 2017).

*P. syringae* pv. tomato strain DAPP-PG 215 was obtained by isolation from an individual lesion on a diseased tomato plant, randomly selected from a field with tomato cultivar ‘Erminia F1’ (Petoseed) in Pontenure (Piacenza, Italy) in 1995 by Buonaurio et al. (1996) and described as race 0 following inoculation on resistant Ontario 7710 plants. In this study, the sequencing and genome analysis of this strain was performed and, according to the *in silico* results, we propose a different base for *P. syringae* pv. tomato race determination.

## 2 Materials and methods

### 2.1 Bacterial strain and growth conditions

*Pseudomonas syringae* pv. tomato strain DAPP-PG 215 belongs to the bacterial collection of the Plant Protection Unit, Department of Agricultural, Food and Environmental Sciences, University of Perugia, Italy, where it is stored in a -80°C ultrafreezer as glycerol stocks in 50% (*vol*/*vol*) King’s broth (King et al., 1954). For genome sequencing, strain DAPP-PG 215 was transferred on Luria-Bertani (LB) agar plates (Miller, 1972) and incubated at 28°C overnight; then, an isolated colony was streaked onto the same medium and grown in the same manner. Bacterial strains used for screening were instead grown on nutrient agar (NA) plates and then incubated at 27°C for 12 h.

### 2.2 Whole genome sequencing, assembly, and annotation

For genomic DNA (gDNA) extraction, *P. syringae* pv. tomato strain DAPP-PG 215 was inoculated in LB broth (Miller, 1972) from single colony of a pure bacterial culture on a LB plate and incubated overnight at 28°C. The following day, whole gDNA extraction was performed using the NucleoSpin tissue kit (Macherey-Nagel, Düren, Germany) according to the manufacturer’s protocol with the following specifications: elution buffer was preheated at 70°C before use, and the gDNA was eluted in 60 μL. The gDNA was quantified using a high sensitivity double-stranded DNA (dsDNA) assay (DeNovix, Wilmigton, DE) with a Fluo-100B fluorometer (Allsheng, Hangzhou, China).

Library preparation for short-read sequencing was done using the Nextera XT DNA library prep kit (Illumina, San Diego, CA) following the manufacturer’s instructions. Sequencing was performed on a MiSeq Illumina sequencer with 2 × 300-bp paired-end reads using a MiSeq reagent kit version 3 (Illumina, San Diego, CA) according to the manufacturer’s instructions.

For long-read sequencing, the Gentra PureGene Yeast/Bact kit protocol (Qiagen, Hilden, Germany) was used on an overnight bacterial culture for the gDNA extraction. The gDNA was quantified as described above. Library preparation and sequencing were performed with the ligation sequencing kit (catalog no. SQK-LSK109; Oxford Nanopore Technologies, Oxford, United Kingdom) and run on an R9.4.1 Flongle Flow Cell with a MinION sequencer. The native barcoding expansion kit (catalog no. XP-NBD114; Oxford Nanopore Technologies, Oxford, United Kingdom) was used for multiplexing. Base calling was performed using Guppy version 5.0.11.

A hybrid assembly using the MiSeq and MinION reads was conducted with Unicycler version 0.4.9 (Wick et al., 2017). The genome was then annotated using Bakta version 1.2.4 (Schwengers et al., 2021) and the database version 3.0. All tools were run with default parameters unless otherwise specified. The BioCircos tool (Cui et al., 2016) was used to have a circular visualization of genomic data.

### 2.3 Identification of mobile regions

The genome of *P. syringae* pv. tomato strain DAPP-PG 215 was checked for the presence of potential prophage regions using the search tool PHASTER (Zhou et al., 2011; Arndt et al., 2016). It was thus possible to identify the prophage regions contained in the genome and to have them classified according to their level of completeness into incomplete, questionable, and intact. Genomic island (GI) prediction was carried out using IslandViewer 4 (Bertelli et al., 2017). The homology search of the proteins localized in these regions was performed by BLAST+ v. 2.13.0 (Camacho et al., 2009) using the National Center for Biotechnology Information (NCBI) database.

### 2.4 Selection of genome sequences and comparative analysis

Comparative genome analyses were performed on a total of 72 genomes taken from the *P. syringae* species complex and representatives of seven phylogroups according to Berge et al. (2014), plus *P. syringae* PDD-32b-74 and *P. syringae* pv. tomato DAPP-PG 215 (**Table S1**). Genome sequences were taken from the NCBI RefSeq database (http://www.ncbi.nlm.nih.gov) and added to an EDGAR 3.0 database (Dieckmann et al., 2021). Through the EDGAR platform, the core genome phylogenetic tree was constructed. Briefly, for the core genome of the 74 selected genomes, the EDGAR pipeline made an alignment for each of the 1,969 gene sets of the core genome using the MUSCLE software (Edgar, 2004). The resulting alignments for 145,706 genes in total were then concatenated into one multiple alignment of 753,574 AA-residues per genome, 55,764,476 AA-residues in total. This large alignment was submitted as input to FastTree 2 software (Price et al., 2010) within EDGAR 3.0, which processed a maximum likelihood phylogenetic tree and verified the tree topology using the Shimodaira-Hasegawa test.

**Figure 1:**
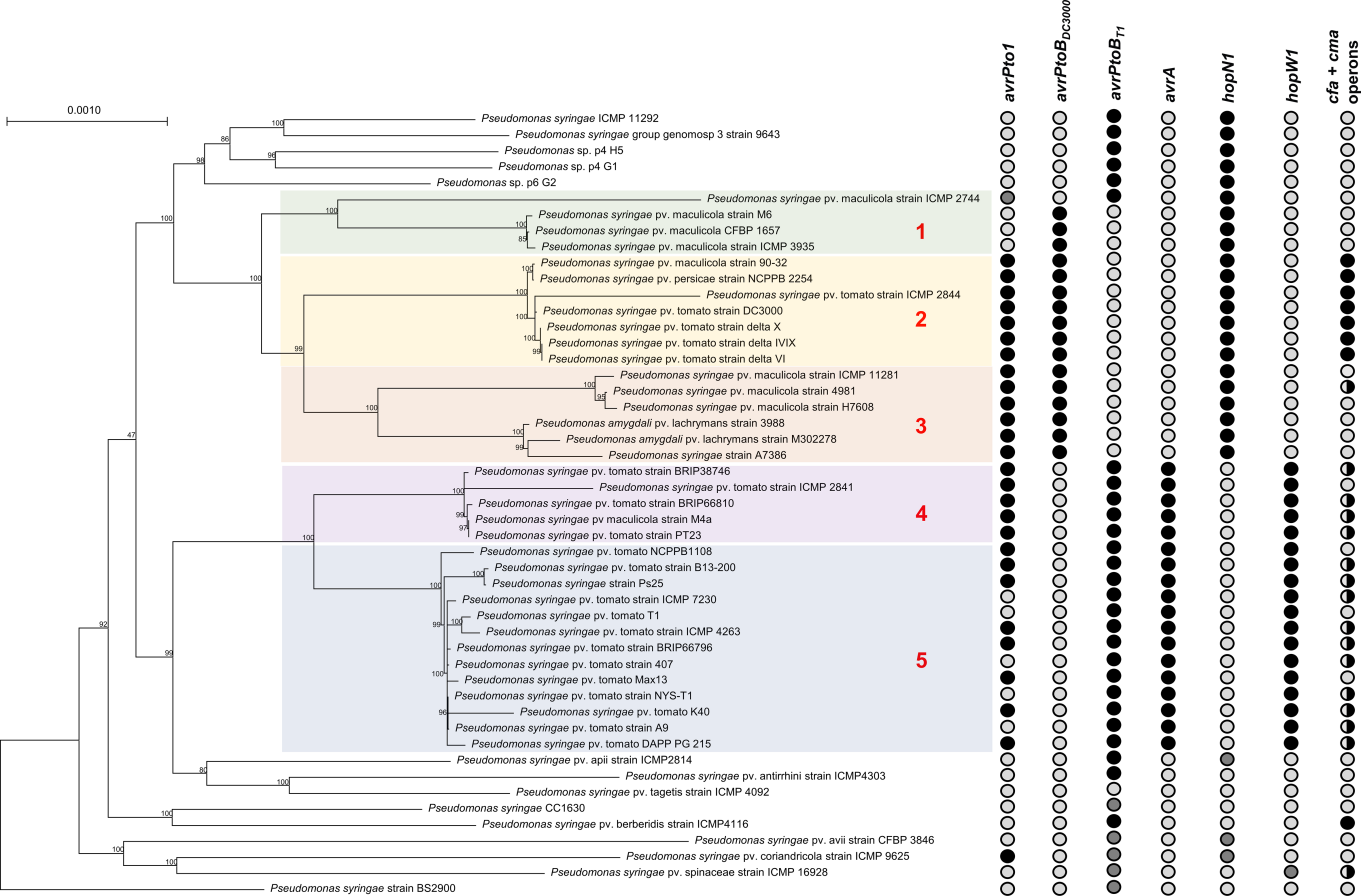
Subgroups defined according to core phylogeny for later comparative analysis. The green branch represents the first subgroup; the yellow branch represents the second subgroup; the orange branch represents the third subgroup; the pink branch represents the fourth subgroup; the blue branch represents the fifth subgroup. Dots in the right part of the figure are meant to show the absence or the presence of avirulence (*avr*) and Hrp-dependent outer protein (*hop*) genes *avrPto1*, *avrPtoB_DC3000_*, *avrPtoB_T1_*, *avrA*, *hopN1* and *hopW1*, and of coronatine synthesis genes belonging to the *cfa* and *cma* clusters. Dot filling indicates the presence with high sequence identity (black), the presence with low sequence identity (dark grey) and absence of the genes (white); half-filled dots for the coronatine genes indicates presence of these genes with a plasmid origin.

Based on the phylogenetic tree obtained by comparing the core genomes of all strains in **Table S1**, the genomes belonging to PG01a were divided into five different subgroups (**Figure 1**). Using the subroutine in EDGAR 3.0, five meta-core genomes representing the core genome of all strains included in each subgroup were defined. Subsequently, the EDGAR pipeline was used to perform a comparison of the different meta-core genomes, in order to reveal the presence of orthologous genes for each subgroup. Protein sequences resulting from all the comparisons were then displayed in a Venn diagram.

### 2.5 Distribution of avirulence gene *avrPto1* and *avrPtoB*

To test whether there was a different distribution of the *avrPto1* and *avrPtoB* genes in the selected genomes, a pan-genome analysis of all strains was performed in EDGAR 3.0. Annotation gaps were checked using BLASTX (Altschul et al., 1990) and compared to a database of bacterial reference proteins (Refseq_proteins) restricted to “*Pseudomonas syringae* group (taxid:136849)”. Standard settings were used for all other parameters. Nucleotide sequences corresponding to each identified gene were used to analyze the phylogenetic relationship existing between the selected genomes and those containing *avrPto1* and *avrPtoB* genes. The resulting phylogenetic trees were carried out with Molecular Evolutionary Genetics Analysis v11 (MEGA11) software (Tamura et al., 2021), according to the neighbor-joining method (Saitou & Nei, 1987). To model rates amongst sites, a gamma distribution (*α* = 5) was used and a partial deletion consisting in a 95% cut-off of the site coverage was applied to gaps and missing data. Furthermore, alignments from the MUSCLE algorithm were also checked for protein identity and coverage with NCBI Multiple Sequence Alignment Viewer 1.22.2 (MSA) using *P. syringae* pv. tomato DAPP-PG 215 sequences as anchor.

The presence of potential prophages was also assessed for all the strains carrying the gene *avrPto1* using PHASTER (Zhou et al., 2011; Arndt et al., 2016) and visualized with MAUVE v2.4.0 (Darling et al., 2004).

### 2.6 Data availability

The genome sequence of *P. syringae* pv. tomato DAPP-PG 215 were submitted to EMBL and received the Assembly accession number GCA_949769235. Additional genome sequences analyzed within this study are available in the NCBI GenBank/DDJ/EMBL database under the accession numbers detailed in **Table S1**. The original contributions presented in the study are publicly available. This data can be found here: NCBI, PRJEB59188, OX458335-7.

## 3 Results

### 3.1 Whole genome general features

**Figure 2:**
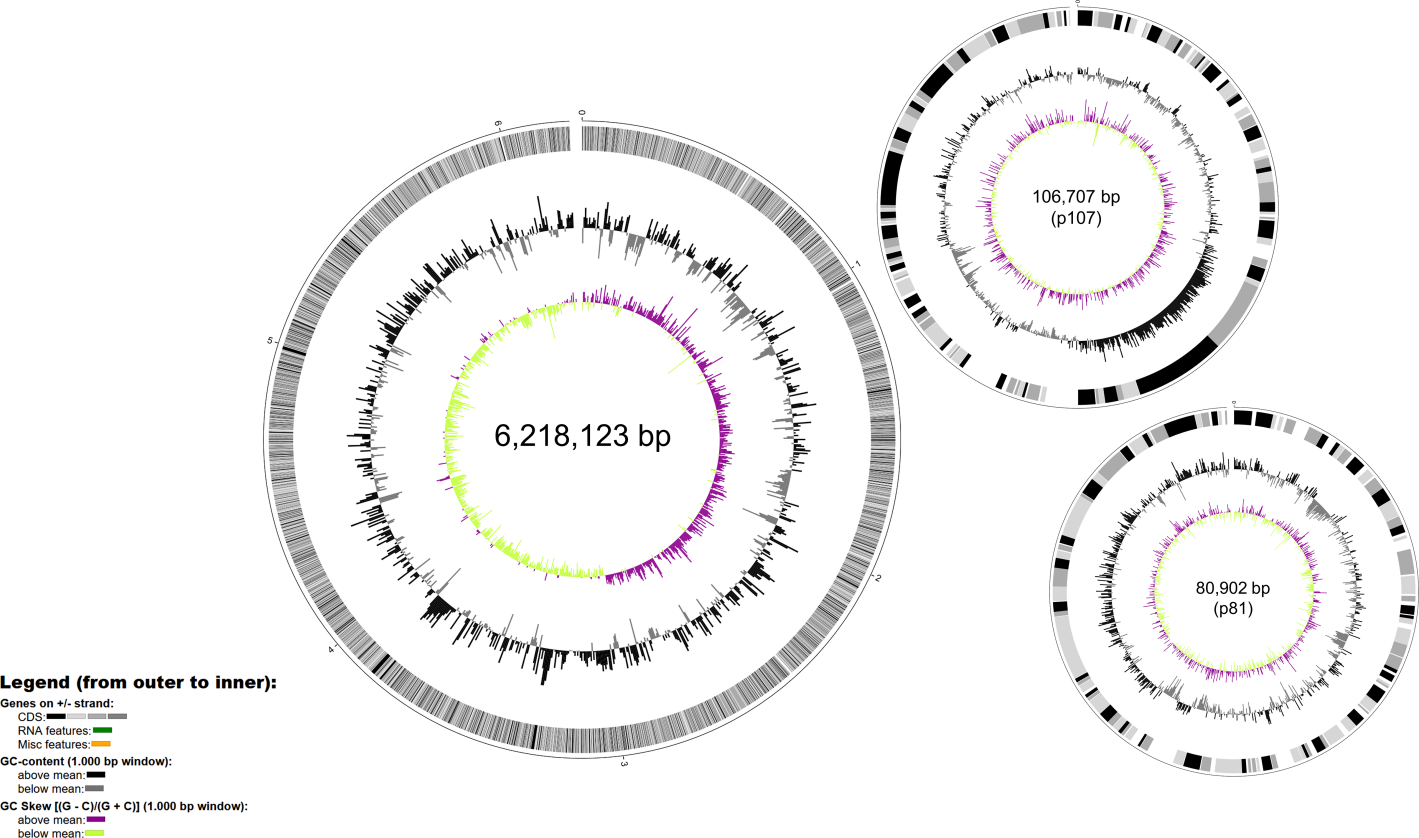
Map of the genome of *Pseudomonas syringae* pv. tomato DAPP-PG 215 generated with BioCircos. In the outermost ring, identified CDS are in grey, RNA features in green and miscellaneous features are in orange. In the intermediate ring GC content in grey and the innermost ring represents the GC skew above the mean in purple and GC skew below the mean in green.

The complete genome of *P. syringae* pv. tomato DAPP-PG 215 consists of a 6,218,123 bp circular chromosome and two circular plasmids of 106,705 bp (p107) and 80,902 bp (p81), with a GC content of 59%, 58% and 57%, respectively (**Figure 2**). The 6.2 Mb chromosome comprises of 5,595 protein coding sequences (CDS), while plasmids p107 and p81 have 103 and 92 CDS, respectively. In the whole genome, 642 CDS were identified as hypothetical proteins, and 72 predicted tRNAs and 5 rRNA operons were found. Among the most prominent genomic features for the characterization of strain DAPP-PG 215 is the presence of the avirulence genes *avrPto1* and *avrPtoB*, but also of the effector genes *hopW1* and *avrA*, which are considered as diagnostic markers for race 1 (Jones et al., 2015), while the *hopN1* gene (a diagnostic marker for race 0 strains), was missing (**Figure 1**). Gene clusters for the biosynthesis of coronafacic acid and coronamic acid, precursors of the phytotoxin coronatine, *hopK1* and a type VI Hcp1 effector gene were detected within plasmid p107, while in plasmid p81, effector genes *avrD1*, *hopD1* and *hopQ1-1* were found.

Analysis of the DNA mobile regions using PHASTER indicated a total of nine prophages in the chromosome and two in plasmid p107. Information related to their size and their completeness level can be found in **Table S2**. Most of the prophages detected were taxonomically similar to phage families belonging to the class *Caudoviricetes* such as *Myoviridae*, *Podoviridae* and *Siphoviridae* (Iwasaki et al., 2018). Within prophage region 9 (PR-9, from position 5,843,821 to 5,884,716), the *avrPto1* gene is located. Genomic islands (GIs), clusters of genes obtained by horizontal gene transfer, were also found in both the chromosome and the plasmids of strain DAPP-PG 215. In the chromosome, a total of 44 GIs were detected by IslandViewer 4 (with the SIGI-HMM prediction method); while two and a single GIs were predicted for plasmids p107 and p81, respectively (**Table S2**). Most of the GIs contained transposases, integrases, phage-related genes and hypothetical protein-coding genes. However, genes for protein effectors associated with the type III secretion system were annotated within the first GI on plasmid p81 and in six GIs on the chromosome. More specifically, effector genes *hopD1* and *hopQ1-1* within both GI-10 and GI-46, *hop-T1* in GI-13, *hopF2* in GI-16, *hopC1* in GI-23, *hopAF1* in GI-32, *hopT1-2* and *hopS* in GI-35 were predicted. Genes associated with arsenic resistance such as *arsC* and *arsN1* were found within GI-6, while in GI-4 the algorithm detected the presence of two fluoride exporters (*eriC* and *crcB*).

### 3.2 Phylogenomic analysis

Phylogenomic analysis conducted using the core genomes of the 74 strains listed in **Table S1** has confirmed the correctness of the phylogroup clustering made by Berge et al. (2014) and revealed that strain DAPP-PG 215 is part of the PG01a, as well as the other *P. syringae* pv. tomato strains used for the comparison (**Figure S1**). Furthermore, looking more closely at the clustering of PG01a, it is possible to see that the *P. syringae* pv. tomato strains are divided into two different subgroups: in the first group, race 0 strain DC3000 was found along with strain ICMP 2844, *P. syringae* pv. persicae NCPPB 2254, *P. syringae* A7386 and a few strains of *P. syringae* pv. maculicola and *P. amygdali* pv. lachrymans while in the second group, several known strains of *P. syringae* pv. tomato race 1 such as strains T1, NYS-T1, Max13, K40 and NCPPB 1108, but also *P. syringae* pv. maculicola M4a, were identified. Strain DAPP-PG 215 was included within the second subgroup. This finding confirms again the greater genotypic proximity of this strain to race 1 P*. syringae* pv. tomato strains.

### 3.3 Comparative genomics

**Figure 3:**
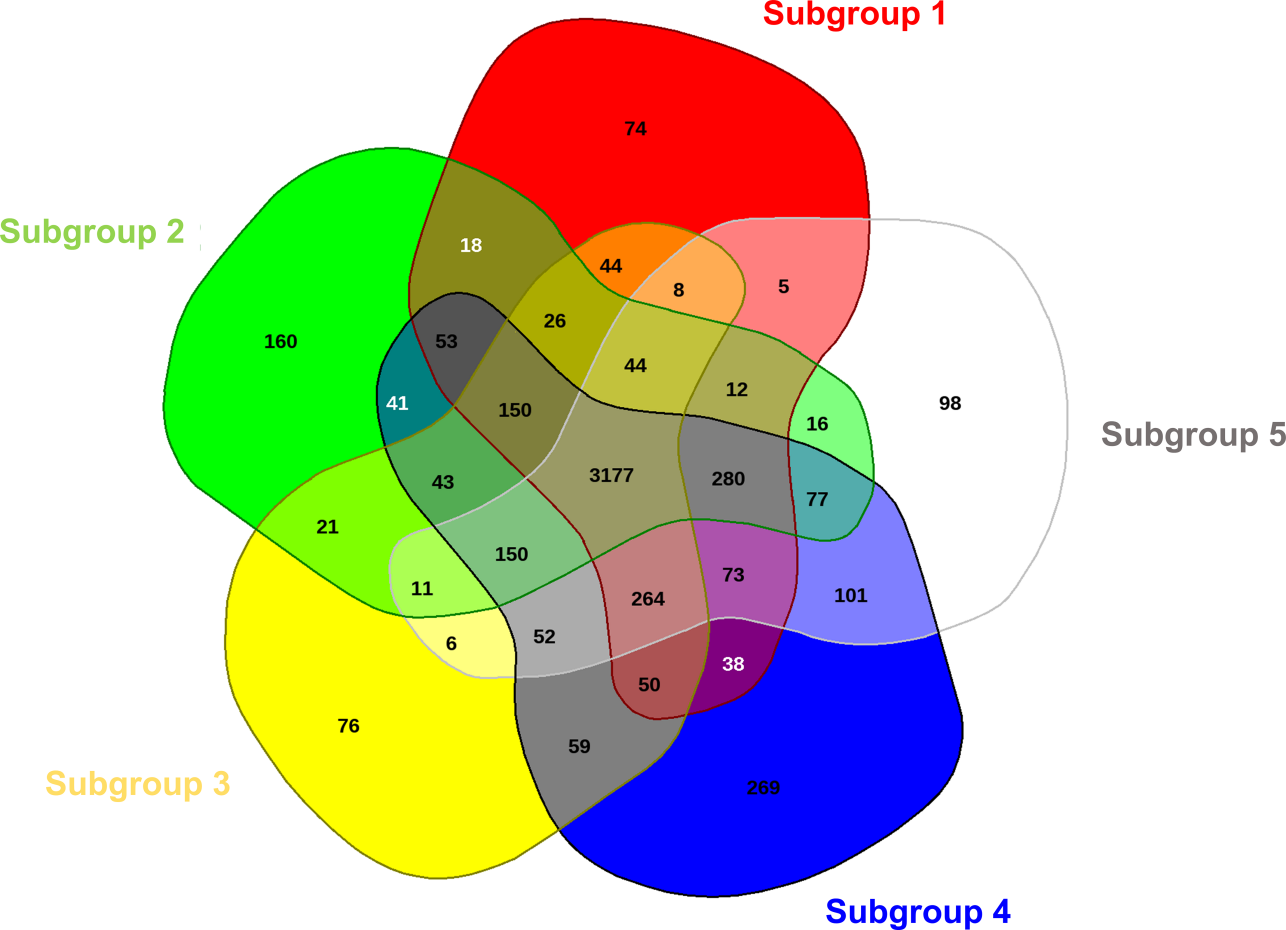
Comparative analysis of the meta-core-genomes of five different defined groups in PG01a. The Venn diagram, constructed with EDGAR 3.0, shows unique and shared genome components for all the five groups.

By comparing the five representative meta-core genomes of the subgroups defined in **Figure 1**, it was possible to identify which and how many orthologous genes were shared among the different subgroups and which genes were instead distinctive for each of them (**Figure 3**). A total number of 5,496 CDS were retrieved from the analysis, of which 3,177 (57.8%), 1,642 (29.9%) and 677 (12.3%) were assigned as core genome, disposable and singleton components, respectively. Among the latter, the distinctive proteins for each subset ranged from 74 (subgroup 1) to 269 (subgroup 4). Mostly, the singletons for each subgroup were hypothetical proteins and transposable elements. However, within the core genome of specific subgroups, distinctive genes were also found coding for: (a) the virulence effectors HopAD1 and HopK1, a TonB-dependent siderophore receptor, the coronafacic acid biosynthetic enzymes, and the proteins for biosynthesis of a putative bacteriocin in subset 2; (b) the virulence effector HopO1-3 in subgroup 3; (c) various ABC transporters, arsenic-resistance proteins (ArsB, ArsC, ArsH), the virulence effector AvrPphD, and colicin immunity protein in subset 4; and (d) copper resistance proteins (CopB, CopC and CopD) in subset 5. It is important to mention as well that the *avrPtoB* gene was observed to be one of the 3,177 core genes shared among all the subgroups.

### 3.4 Avirulence gene *avrPto1* is located in a prophage

Since the *avrPto1* gene in *P. syringae* pv. tomato DAPP-PG 215 was found within an intact prophage region (PR-12, *Pseudomonas* phage *phi3*), the same analysis was also performed on all genomes that were part of the subgroups defined above (**Figure 1**). For all strains having the *avrPto1* gene, PHASTER detected the presence of *Pseudomonas* phage *phi3* prophage in the genome (**Table 1**). Despite the different degree of completeness of the region, for all strains, it was in a position overlapping with that of the *avrPto1* gene. Strains lacking *avrPto1* such as *P. syringae* pv*. tomato* race 1 strains A9, T1, NYS-T1, BRIP 66796 and 407, on the other hand, also did not have *Pseudomonas* phage *phi3*. It also appears that this *avrPto1*-containing prophage is located at different locations in the genome. Whereas in *P. syringae* pv. tomato DAPP-PG 215, it is inserted in the chromosome, between another prophage (Vibrio_vB_VpaM_MAR_NC_019722) and two contiguous transposons of the IS*Psy4* family transposase, flanked by *attL* and *attR* sites of 11 bp (CCACGTAACAAG), in *P. syringae* DC3000, it is located in the chromosome as well, but about 1.35 Mb upstream of the attachment site *attL* of *P. syringae* pv. tomato DAPP-PG 215, flanked by different 11 bp *attL* and *attR* sites (CCTGACGATGAA).

**Table 1:** PHASTER predictions of prophage Pseudo_phi3_NC_030940 locations in all *Pseudomonas syringae* pv. tomato strains used in this study and all the PG01a strains carrying avrPto1 in comparison with avrPto1 locations for each of these strains.

		*PHAGE_Pseudo_phi3_NC_030940*			*avrPto1*	
Subgroup	Strain	+/-	Locus	Region	Locus	Region
1	*P. syringae* pv. maculicola ICMP 2744	+	NZ_RBQA01000301.1	108-35161	NZ_RBQA01000301.1	1135-1635
2	*P. syringae* pv. maculicola 90-32	+	LGLH01000007.1	41073-62385	LGLH01000007.1	40462-40956
2	*P. syringae* pv. persicae NCPPB 2254	+	LAZV01000025.1	1-23807	LAZV01000025.1	1547-2041
2	*P. syringae* pv. tomato ICMP 2844	+	LJRN01000253.1^1^	1-8438^1^	LJRN01000253.1	1099-1593
2	*P. syringa*e pv. tomato DC3000	+	NC_004578.1	4505488-4530373	NC_004578.1	4514766-4515260
2	*P. syringae* pv. tomato delta X	+	NZ_CP047073.1	4505480-4530365	NZ_CP047073.1	4514758-4515252
2	*P. syringae* pv. tomato delta IV, IX	+	NZ_CP047072.1	4505481-4530366	NZ_CP047072.1	4514759-4515253
2	*P. syringae* pv. tomato delta VI	+	NZ_CP047071.1	4505477-4530362	NZ_CP047071.1	4514755-4515249
3	*P. syringae* pv. maculicola ICMP 11281	+	NZ_RBUQ01000113.1	274-18458	NZ_RBUQ01000164.1	1061-1555
			NZ_RBUQ01000164.1^2^			
3	*P. syringae* pv. maculicola ICMP 4981	+	NZ_RBOO01000069.1	35873-59467	NZ_RBOO01000069.1	37554-38048
3	*P. syringae* pv. maculicola H7608	+	NZ_LGLG01000401.1	10054-33648	NZ_LGLG01000401.1	11735-12229
3	*P. amygdali* pv. lachrymans 3988	+	NZ_LGLJ01000049.1	83427-129946	NZ_LGLJ01000049.1	94628-95122
3	*P. amygdali* pv. lachrymans M302278	+	NZ_GL385206.1	88977-127163	NZ_GL385206.1	91352-91846
3	*P. syringae* A7386	+	NZ_RBOB01000082.1	2233-36720	NZ_RBOB01000082.1	33794-34288
4	*P. syringae* BRIP38746	+	NZ_SNVG01000016.1	73880-108746	NZ_SNVG01000016.1	76255-76749
4	*P. syringae* pv. tomato ICMP 2841	+	NZ_RBUK01000087.1	3203-24750	NZ_RBUK01000087.1	9618-10112
4	*P. syringae* pv. tomato BRIP66810	+	NZ_SNVE01000036.1	1-34047	NZ_SNVE01000036.1	1556-2050
4	*P. syringae* pv. maculicola M4a	+	NZ_LGLE01000008.1	1006002-1040868	NZ_LGLE01000008.1	1008377-1008871
4	*P. syringae* pv. tomato PT23	+	NZ_MSDS01000023.1	464-35330	NZ_MSDS01000023.1	2839-3333
5	*P. syringae* pv. tomato NCPPB 1108	+	NZ_ADGA01000002.1	7121-41975	NZ_ADGA01000002.1	39117-39611
5	*P. syringae* pv. tomato B13-200	+	NZ_CP019871.1	5938347-5973213	NZ_CP019871.1	5940722-5941216
5	*P. syringae* Ps25	+	NZ_CP034558.1	926805-962874	NZ_CP034558.1	960016-960510
5	*P. syringae* pv. tomato ICMP 7230	+	NZ_RBRI01000207.1	439-35293	NZ_RBRI01000207.1	32435-32929
5	*P. syringae* pv. tomato T1	–	–	–	–	–
5	*P. syringae* pv. tomato ICMP 4263	+	NZ_RBRJ01000006.1	439-35293	NZ_RBRJ01000006.1	32435-32929
5	*P. syringae* pv. tomato BRIP66796	–	–	–	–	–
5	*P. syringae* pv. tomato 407	–	–	–	–	–
5	*P. syringae* pv. tomato Max13	+	NZ_ADFZ01000218.1	21015-55880	NZ_ADFZ01000218.1	23390-23884
5	*P. syringae* pv. tomato NYS-T1	–	–	–	–	–
5	*P. syringae* pv. tomato K40	+	NZ_ADFY01000134.1	280-21260	NZ_ADFY01000134.1	2485-2979
5	*P. syringae* pv. tomato A9	–	–	–	–	–
5	*P. syringae* pv. tomato DAPP-PG 215	+	GCA_949769235	5852865-5887730	GCA_949769235	5855240-5855734
–	*P. syringae* pv. coriandricola ICMP 9625	+	NZ_RBRV01000199.1	2567-25399	NZ_RBRV01000199.1	1956-2450

^1^Prophage presence for this strain was identified with MAUVE software.

^2^Part of the prophage in this strain was found in NZ_RBUQ01000113.1, and the other in NZ_RBUQ01000164.1 (MAUVE software).

### 3.5 Distribution of the avirulence gene *avrPto1* in genome sequences

A BLASTX search was performed using as queries the *avrPto1* and *avrPtoB* sequences of *P. syringae* pv. tomato DAPP-PG 215 obtained with the aim of determining the evolutionary relationship existing between this specific strain and the other strains included in PG01a concerning these two avirulence genes. The BLASTX analysis revealed the presence of *avrPto1* in 28 out of 49 strains within PG01a, while *avrPtoB* was found in 46 out of 49 genomes.

Comparing the dendrogram based on the presence or absence of *avrPto1* with the core genome phylogeny, it was observed that although some of the major subgroups defined in PG01a by the phylogenetic analysis were confirmed, their positioning in the structure of these trees was not consistent (**Figure S2**). For AvrPto1, the sequence of *P. syringae* pv. tomato DAPP-PG 215 was found to cluster with those of strains of subgroup IV and V, in a branch of the tree that was only slightly distinct from subgroup II (98.82% protein identity, due to two substitutions: V49L and Y156A) and a part of subgroup III (*P. syringae* A7386 and *P. amygdali* pv. lachrymans strains 3988 and M302278), while the *P. syringae* pv. maculicola strains from subgroup III (ICMP 11281, 4981 and H7608, respectively) clustered together more distantly from the two groups (68.67% protein identity). The only strain of subgroup I carrying *avrPto1* was *P. syringae* pv. maculicola ICMP 2744, the protein sequence of which was very distant to the AvrPto1 sequences of the other strains mentioned above (33.94% protein identity with *P. syringae* pv. tomato DAPP-PG 215). An AvrPto1 ortholog was also found in *P. syringae* pv. coriandricola ICMP 9625 (96.45% protein identity with *P. syringae* pv. tomato DAPP-PG 215). Among the five amino acid changes between the AvrPto1 protein sequences of *P. syringae* pv. coriandricola ICMP 9625 and *P. syringae* pv. tomato DAPP-PG 215 (I4M, D29G, A44S, Y90F and G97R), the fifth is occurring inside the GINP loop, a region known to be responsible for the interaction with plant Pto (Shan et al., 2000; Chang et al., 2001; Wulf et al., 2004). Lastly, it was observed that, unlike subgroups II and III, the *avrPto1* gene was not observed in subgroups I and V: *P. syringae* pv. tomato race 1 strains T1, ICMP 7230, 407, NYS-T1 and A9 and *P. syringae* pv. maculicola strains M6, CFBP 1657 and ICMP 3935 were all lacking it.

### 3.6 Distribution of the avirulence gene *avrPtoB* in genome sequences

For orthologs of *avrPtoB*, there are two distinct major clades in the dendrogram (**Figure S3**). In the first clade, gene sequences from all strains included in the subgroups I, II and III were clustering together. However, subgroup III was divided with the *P. syringae* pv. maculicola strains and the *P. syringae* A7386 and *P. amygdali* pv. lachrymans3988 and M302278 positioned in two different parts of the subtree. In the other cluster, gene sequences from strains of subgroups IV and V were clustering together alongside *P. syringae* ICMP 11292, *P. syringae* group genomosp. 3 strain 9643, *Pseudomonas* sp. p4.H5, *Pseudomonas* sp. p4.G1, *Pseudomonas* sp. p6.G2, *P. syringae* pv. antirrhini ICMP 4303, *P. syringae* pv. apii ICMP 2814, *P. syringae* BS2900 and *P. syringae* CC1630. The tree also showed that strain *P. syringae* pv. avii CFBP 3846 also has an ortholog of *avrPtoB*, but it was found to be quite distinct from all other *avrPtoB* gene sequences. The existence of two major groups was consistent with previous discovery of *P. syringae* pv. tomato DC3000 *avrPtoB* orthologs with lower sequence identity in *P. syringae* pv. tomato PT23 (*avrPtoB_PT23_*) and T1 (*avrPtoB*_T1_) strains (Lin et al., 2006). The clear separation of race 0 and race 1 strains could again not be confirmed, as the *avrPtoB* sequence of *P. syringae* pv. tomato DAPP-PG 215 was observed to cluster with *P. syringae* pv. tomato T1 and PT23, rather than with race 0 strain *P. syringae* pv. tomato DC3000. From the amino acid alignment, *avrPtoB* of *P. syringae* pv. tomato DAPP-PG 215 resulted to have an almost identical amino acid sequence as the proteins of *Pseudomonas* sp. p6.G2 (99.64%), *P. syringae* pv. berberidis ICMP4116 and subgroup IV strains (99.29%); while its identity with the other major cluster with subgroups I, II and III strains was around 72.83%. Lower amino acid sequence identities were found with the AvrPtoB orthologs from *P. syringae* CC1630 (58.36% identity, 75.09% coverage), *P. syringae* BS2900 (36.30% identity, 75.98% coverage) and *P. syringae* pv. avii CFBP 3846 (44.84% identity, 89.32% coverage).

### 3.7 Localization of coronatine genes is divergent within PG01a

Coronatine (COR) is a phytotoxin produced by several pathovars of *P. syringae*, and consists in the conjunction of coronafacic acid (CFA) and coronamic acid (CMA), synthetized by the homonymous clusters. Because the presence of the *cfa* and *cma* clusters in plasmids has been observed in different pathovars of *P. syringae* (Bender et al., 1991), we made a comparison among the PG01a strains gene sequences of the *cfa* cluster (*cfa1*-*9*, and the *cfl* gene coding for coronofactate ligase), the *cma* cluster (the *cmaABCDET* cluster and *cmaU*) and the COR regulatory region (*corRSP*). Phylogenetic analysis of the sequences for these twenty genes, obtained using EDGARs ortholog retrieval function, allowed us to observe two different PG01a strains having the necessary clusters for coronatine production groups (**Figure S4**). Resuming the division made previously above (**Figure 1**), a first group includes all the strains from subgroup 4 except *P. syringae* pv. tomato ICMP 2841 and all the strains from subgroup 5 except *P. syringae* pv. tomato NCPPB 1108, T1 and Max13, but also *P. syringae* pv. maculicola ICMP 4891 (subgroup 3) and *P. syringae* pv. spinaceae ICMP 16928. A second group, on the other hand, contains the entire subgroup 2, and *P. syringae* pv. berberidis ICMP 4116. More specifically, the nucleotide sequences of *P. syringae* pv. tomato DAPP-PG 215 have approximately 96% identity with the group containing *P. syringae* pv. tomato DC3000 (90% with *P. syringae* pv. maculicola 90-32), 90% with *P. syringae* pv. berberidis ICMP 4116, 98% with *P. syringae* pv. maculicola ICMP 4891, and 99% with *P. syringae* pv. spinaceae ICMP 16928. Significant deletions (>50 bp) were observed in some genes carried by *P. syringae* pv. tomato K40 (*cfa*6, *cfl*, *cmaT* and *corR*), *P. syringae* pv. tomato NYS-T1 (*cfa6*, *cmaT* and *corR*), *P. syringae* pv. maculicola 90-32 (*cfa4* and *cmaT*) and *P. syringae* pv. maculicola M4a (*cfa4* and *cmaT*). In addition, it was possible to observe that both clusters plus the COR regulatory region were located on plasmid regions regarding strains *P.* syringae Ps25 (pPs252, RefSeq: NZ_CP034559.1) and P. syringae pv. tomato B13-200 (pB13-200A, RefSeq: NZ_CP019872.1). Moreover, checking the sequence upstream of cfl, the presence of a CorR-binding site identical to that found in *P. syringae* pv. glycinea PG4180 (Liyanage et al., 1995; Peñaloza-Vázquez & Bender, 1998) and considered to be essential for transcription of genes involved in coronatine synthesis was observed in *P. syringae* pv. tomato DAPP-PG 215.

## 4 Discussion

*P. syringae* pv. tomato DAPP-PG 215 was described as a race 0 strain (Buonaurio et al., 1996) based on its phenotype expressed upon inoculation on susceptible tomato plants cv. ‘Bonny Best’ (+/+) and resistant cv. ‘Ontario 7710’ (*pto*/*pto*). From the analysis of its genome reported here, it rather possesses the marker genes *avrA* and *hopW1* used to distinguish race 1 from race 0 strains, and instead lacks the *hopN1* gene, which is characteristic for race 0 strains (Jones et al., 2015). The presence of a race 1 genotypic background for *P. syringae* pv. tomato strain DAPP-PG 215 is also supported by the phylogenomic analysis of its core genome in comparison with other PG01a strains, in which it clustered with strains described as race 1, such as *P. syringae* pv. tomato T1 rather than with strains described as race 0, such as *P. syringae* pv. tomato DC3000, and by the sequence analysis of the *avrPtoB* gene, found to be identical to that possessed by strain T1.

Resistance to bacterial speck disease caused by *P. syringae* pv. tomato is due to the occurrence of the *pto* gene in the tomato plant. This genes encodes the protein Pto that is involved in recognition of the type III effectors AvrPto1 and AvrPtoB (HopAB2) expressed by the pathogen (Salmeron et al., 1996; Lin & Martin, 2007). Although it was long assumed that the presence or absence of the two genes was sufficient to distinguish a strain of *P. syringae* pv. tomato race 0 from a race 1, it was recently observed that specific mutations in the *avrPto1* gene prevent the AvrPto1 protein effector from Pto-mediated recognition (Xing et al., 2007). On the other hand, some strains expressing a race 1 phenotype, such as *P. syringae* pv. tomato T1, are capable to evade recognition because they have orthologous *avrPtoB* genes that are weakly transcribed (Lin et al., 2006; Kunkeaw et al., 2010). However, an intact *avrPto1* avirulence gene was also found in *P. syringae* pv. tomato DAPP-PG 215, which could be responsible for the expression of the target of Pto and therefore the reason for the race 0 phenotype expressed by this strain. This hypothesis is in agreement with what was reported by Kraus et al. (2017) who observed the presence of *avrPto1* in some strains of *P. syringae* pv. tomato race 1 and noticed in these strains a growth profile *in planta* intermediate between a race 0 and a race 1. According to what was reported so far, therefore, *P. syringae* pv. tomato DAPP-PG 215 would result in a race 1 genotype and a race 0 strain for its expressed phenotype.

In addition, the detection of the *avrPto1* gene within a prophage in *P. syringae* pv. tomato DAPP-PG 215 and in all other strains belonging to PG01a used in this work possessing this avirulence gene was only reported in this study. Prophages (viral DNA integrated within bacterial genome) are mobile genetic elements that can be responsible for horizontal gene transfer in bacteria. The presence within prophages of genes encoding for virulence effectors of the bacterium is well documented (Fortier & Sekulovic, 2013; Varani et al., 2013), however, since our results suggest that the *avrPto1* gene is associated with this type of mobile regions, the genotype-based criterion for distinguishing race in *P. syringae* pv. tomato appears unstable and unreliable.

Since strains of *P. syringae* pv. tomato having *avrPto1* express a race-typical 0 phenotype even when they carry a low expressed *avrPtoB* homologue such as *avrPtoB*_T1_ (Kraus et al., 2017), it is possible to advance a hypothesis that the phenotype expressed by *P. syringae* pv. tomato will result in a race 1 if the strain lacks a functioning *avrPto1* and instead has the *avrPtoB* ortholog *avrPtoB*_T1_, capable of escaping the Pto recognition, and a race 0 if the *P. syringae* pv. tomato strain meets either of the two following conditions: (a) it lacks a functioning *avrPto1* but has an *avrPtoB*_DC3000_ (Lin et al., 2006), which results in a normal expression of AvrPtoB; (b) it has a functioning *avrPto1*, which, regardless of which *avrPtoB* gene is present in its genome, will be recognized by Pto.

Another difference between strains having a race 0 genomic background and those having a race 1 is the type of coronatine biosynthesis genes. Coronatine is a phytotoxin that interferes with stomata closure in response to pathogen-associated molecular patterns (PAMPs) and salicylic acid-mediated plant defense, also contributing to the development of chlorotic spots on plant leaves (Geng et al., 2014). Produced by strains of various pathovars of *P. syringae*, such as the pathovars atropurpurea, glycinea, maculicola, morsprunorum, and tomato (Bereswill et al., 1994), the genes involved in its synthesis are either located on plasmids or integrated in the chromosome (Preston, 2000). More specifically, the *cfa* and *cma* clusters, required for the synthesis of coronafacic acid and coronamic acid, respectively, and the regulatory region separating them containing the three genes *corP*, *corS*, and *corR*, are found in the chromosome in several strains of *P. syringae* pv. maculicola (including ICMP 2744) and in *P. syringae* pv. tomato DC3000, while in other strains such as *P. syringae* pv. tomato PT23 and *P. syringae* pv. maculicola 4981, they are found on a plasmid (Bender et al., 1989; Cuppels & Ainsworth, 1995).

From our analyses, genes for coronatine were also found on a plasmid in strains *P. syringae* Ps25, *P. syringae* pv tomato B13-200, and *P. syringae* pv tomato DAPP-PG 215. In addition, between the latter named strains and *P. syringae* pv. tomato DC3000, the *cfa* and *cma* clusters have about 96% identity at the nucleotide level, a finding in agreement with what was observed by in *P. syringae* pv. glycinea PG4180, which also had the genes for coronatine located in a plasmid (Wang et al., 2002). The subdivision in the phylogenetic analysis showed that all strains having the coronatine genes in the plasmid mentioned earlier clustered in one group, while *P. syringae* pv. tomato DC3000, which has the genes on the chromosome, was part of the other group. Although it has not been possible to verify for *P. syringae* pv. maculicola ICMP 2744 belonging to the same group as *P. syringae* pv. tomato DC3000, it is possible to assume that all strains belonging to the group with *P. syringae* pv. tomato DAPP-PG 215, B13-200, PT23, *P. syringae* Ps25 and *P. syringae* pv. maculicola 4981 have the genes for coronatine located on a plasmid, while *P. syringae* pv. tomato ICMP 2844, *P. syringae* pv. persicae NCPPB 2254 and *P. syringae* pv. maculicola 90-32 have them on the chromosome. Although it is currently unclear what the implications are for having the gene clusters for coronatine located on the plasmid rather than on the chromosome, a different regulatory mechanism for the synthesis of this phytotoxin was proposed for *P. syringae* pv. tomato DC3000 (Fouts et al., 2002), which lacks the CorR-binding site upstream *cfl* (Wang et al., 2002).

The results of this study confirm what has been previously observed regarding the distribution of genes for coronatine in some strains of *P. syringae* pv. tomato and *P. syringae* pv. maculicola and proposes new hypotheses in the distinction between strains carrying the coronatine biosynthetic genes on plasmids or on chromosomes, although molecular investigations are needed to better understand the significance of this difference. The existence of strains of *P. syringae* pv. tomato having an intermediate phenotype between a race 0 and a race 1 is also confirmed. Subsequent studies that can estimate the ability of the respective strains to grow *in planta* and the severity of the disease on tomato plants in comparison with other non-intermediate strains are required to understand the role of *avrPto1* in the various genomic assets. Lastly, the presence of the *avrPto1* gene in a prophage may result in a different awareness of the concept of race in *P. syringae* pv. tomato, which is therefore, on a phenotypic basis, to be intended as potentially less permanent.

Whereas the race concept may thus become obsolete, it still needs to be commented that strains of both races were and will remain pathogens of tomato, irrespective of their genetic background.

## Data availability statement

The datasets presented in this study can be found in online repositories. The names of the repository/repositories and accession number(s) can be found in the article/**Supplementary Material**.

## Author contributions

BO and TS have conceptualized and designed the study. JP has carried out the bacterial cultivation, DNA extraction and NGS sequencing. JP, LF, JB and TS have performed the bioinformatic and software analysis. CM conducted the molecular investigations. RB and TS provided the resources needed for the analysis. BO and TS have prepared the original draft preparation. RB, CM and TS have contributed to the funding acquisition. All authors contributed to the article and approved the submitted version.
