# Supplementary material for: Race-specific genotypes of Pseudomonas syringae pv. tomato are defined by the presence of mobile DNA elements within the genome
Source: Front Plant Sci. 2023 Jul 5;14:1197706. doi: 10.3389/fpls.2023.1197706 (PMC10354423; doi:10.3389/fpls.2023.1197706)
Supplement: Supplementary file 1 [file DataSheet_1.docx]

Supplementary Material

Race-specific genotypes of *Pseudomonas syringae* pv. tomato are defined by the presence of mobile DNA elements within the genome

Benedetta Orfei^1^, Joël F. Pothier^2^, Linda Fenske^3^, Jochen Blom^3^, Chiaraluce Moretti^1,*^, Roberto Buonaurio^1^ and Theo H. M. Smits^2,*^

^1^Dipartimento di Scienze Agrarie, Alimentari e Ambientali (DSA3), Università degli Studi di Perugia, Perugia, Italy

^2^Environmental Genomics and Systems Biology Research Group, Institute of Natural Resource Sciences (IUNR), Zurich University of Applied Sciences ZHAW, Wädenswil, Switzerland

^3^Bioinformatics and Systems Biology, Justus-Liebig University Giessen, Giessen, Germany

*** Correspondence:**Chiaraluce Moretti

[chiaraluce.moretti@unipg.it](mailto:chiaraluce.moretti@unipg.it)

Theo H. M. Smits

[theo.smits@zhaw.ch](mailto:theo.smits@zhaw.ch)

# Supplementary Figures and Tables

**Figure S1.** Core genome phylogenetic tree of 73 genomes taken from the *Pseudomonas syringae* species complex and representative of 10 out of 13 phylogroups according to Berge *et al*. (2014), plus the newly sequenced *P. syringae* pv. tomato DAPP-PG 215. To build the tree, the core genes of the selected genomes are computed. In a following step, alignments of each core gene set are generated using MUSCLE, and the alignments are concatenated to one huge alignment. This alignment of 753,574 AA-residues per genome is then used as input for FastTree software to build the tree. Numbers at the nodes are local support values computed by FastTree using the Shimodaira-Hasegawa test.

**Figure S2.** Dendrogram based on the presence of *avrPto1* in the *Pseudomonas syringae* phylogroup PG01a. Avirulence gene *avrPto1* was identified in 28 genomes out of 49. Dendrogram was constructed with MEGA 11 (Tamura et al., 2021) according to neighbor-joining clustering method (Saitou and Nei, 1987) with gamma distribution (*α* = 5) and 95% cut-off of the site coverage to gaps and missing data partial deletion. Numbers at the nodes are bootstrap values for 1,000 replicates expressed as a percentage value.

**Figure S3.** Dendrogram based on the presence of *avrPtoB* in the *Pseudomonas syringae* phylogroup PG01a. Avirulence gene *avrPtoB* was identified in 46 genomes out of 49. Dendrogram was built with MEGA 11 (Tamura et al., 2021) according to neighbor-joining clustering method (Saitou and Nei, 1987) with gamma distribution (*α* = 5) and 95% cut-off of the site coverage to gaps and missing data partial deletion. Numbers at the nodes are bootstrap values for 1,000 replicates expressed as a percentage value.

**Figure S4.** Concatenated neighbor-joining tree based on *corP*, *corS*, *corR*, and *cfa* (*cfa1*, *cfa2*, *cfa3*, *cfa4*, *cfa5*, *cfa6*, *cfa7*, *cfa8*, *cfa9* and *cfl*) and *cma (cmaA*, *cmaB*, *cmaC*, *cmaD*, *cmaE*, *cmaT*, *cmaU*) cluster gene sequences for all the *Pseudomonas syringae* phylogroup PG01a strains carrying these genes for coronatine synthesis (concatenated alignment length: 90,558 bp). Tree was built with MEGA 11 (Tamura et al., 2021) according to neighbor-joining clustering method (Saitou and Nei, 1987) with gamma distribution (*α* = 5) and 95% cut-off of the site coverage to gaps and missing data partial deletion. Numbers at the nodes are bootstrap values for 1,000 replicates expressed as a percentage value.

**Table S1.** List of genome sequenced *Pseudomonas* strains used in this study.

| **Strain** | **Origin** | **Host** | **GenBank accession** | **Reference** |
| --- | --- | --- | --- | --- |
| *P. cerasi* PL58^T^ | PL, 2007 | *Prunus cerasus* | LT222313-LT222319 | Kałużna *et al*., 2016 |
| *P. syringae* CC1557 | FR, 2006 | Snow | CP007014-CP007015 | N.A. |
| *P. syringae* pv. tomato DAPP-PG 215 | IT, 1995 | *Solanum lycopersicum* | GCA_949769235 | Buonaurio *et al*., 1996 |
| *P. syringae* pv. actinidiae ICMP 9853 | JAP, 1984 | *Actinidia deliciosa* | CP018202-CP018204 | N.A. |
| *P. syringae* pv. tomato DC3000 | UK, 1960 | *Solanum lycopersicum* | AE016853-AE016855 | Buell *et al*., 2003 |
| *P.* syringae pv. tomato B13-200 | CA, 2016 | *Solanum lycopersicum* | CP019871-CP19874 | N.A. |
| *P. syringae* Ps25 |  | *Solanum lycopersicum* | CP034558 | Mazo-Molina *et al*., 2019 |
| *P. amygdali* pv. lachrymans M302278 | US, 1935 | *Cucumis sativus* | AEAM00000000 | Baltrus *et al*., 2011 |
| *P. amygdali* pv. lachrymans 3988 | US, 1935 | *Cucumis sativus* | LGLJ00000000 | N.A. |
| *P. amygdali* ICMP 3918 |  |  | LKBW00000000 | N.A. |
| *P. avellanae* CC1416 | US, 2004 | Epilithon | AVEP00000000 | Baltrus *et al*., 2014 |
| *P. avellanae* JCM 11937 | GRC, 2003 | *Corylus avellana* | BMNO00000000 | N.A. |
| *P. cannabina* ICMP 2823 | HU, 1957 | *Cannabis sativa* | LJPX00000000 | Thakur *et al*., 2016 |
| *P. caricapapayae* CCUG 32775T | BR | *Carica papaya* | VXJY00000000 | N.A. |
| *P. congelans* DSM 14939 |  |  | FNJH00000000 | N.A. |
| *P. coronafaciens* pv. zizaniae ICMP 8959 |  |  | RBPM00000000 | N.A. |
| *P. ficuserectae* ICMP 7849 | JP | *Ficus erecta* | RBSO00000000 | Dillon *et al*., 2019 |
| *P. meliae* CFBP 3225 | JP, 1974 | *Melia azedarach* | JYHE00000000 | Bartoli *et al.*, 2015 |
| *P. savastanoi* 4352 |  | *Olea europaea* | LGKR00000000 | N.A. |
| *Pseudomonas* sp. p4 G1 |  |  | UUPT01000001-UUPT01000064 | N.A. |
| *Pseudomonas* sp. p4 H5 |  |  | UVTG01000001-UVTG01000101 | N.A. |
| *Pseudomonas* sp. p6 G2 |  |  | UVYY01000001-UVYY01000240 | N.A. |
| *P. syringae* CC1559 | FR, 2006 | Snow | AVEG00000000 | Baltrus *et al*., 2014 |
| *P. syringae* CC1630 | US, 2007 | *Onobrychis* sp. | AVED00000000 | Baltrus *et al*., 2014 |
| *P. syringae* ICMP 11292 | NZ, 1991 | *Actinidia deliciosa* | LKGU00000000 | N.A. |
| *P. syringae* KCTC 12500 |  |  | AYTM00000000 | N.A. |
| *P. syringae* group genomosp. 3 strain 9643 | UK, 2012 | *Prunus domestica* | MLET00000000 | N.A. |
| *P. syringae* group genomosp. 3 strain CFBP 3800 |  |  | OLMQ00000000 | N.A. |
| *P. syringae* group genomosp. 3 strain RM1 | UK, 2013 | *Aquilegia vulgaris* | MLEU00000000 | N.A. |
| *P. syringae* pv. actinidifoliorum ICMP18802 | NZ | *Actinidia chinensis* | MUKM00000000 | N.A. |
| *P. syringae* pv. antirrhini ICMP4303 |  |  | LJPT00000000 | N.A. |
| *P. syringae* pv. apii ICMP2814 | US | *Apium graveolens L. var. dulce* | LJPR00000000 | Thakur *et al*., 2016 |
| *P. syringae* pv. avii CFBP 3846 | FR, 1991 | *Prunus avium* | LT963402-LT963407 | Ruinelli *et al.*, 2019 |
| *P. syringae* pv. berberidis ICMP4116 | NZ |  | LJPU00000000 | Thakur *et al*., 2016 |
| *P. syringae* pv. cilantro 0788 9 | US, 1988 | *Coriandrum sativum* | LGLN00000000 | Mott *et al*., 2016 |
| *P. syringae* pv. coriandricola ICMP 9625 | NZ | *Coriandrum sativum* | RBRV00000000 | Dillon *et al*., 2019 |
| *P. syringae* pv. delphinii ICMP 4330 | UK | *Delphinium* sp. | RBQG00000000 | Dillon *et al*., 2019 |
| *P. syringae* pv. maculicola CFBP 1657 | NZ, 1965 | *Brassica oleracea* | JYHH00000000 | Bartoli *et al*., 2015 |
| *P. syringae* pv. maculicola M6 | UK, 1965 | *Brassica oleracea* | LGLC00000000 | N.A. |
| *P. syringae* pv. maculicola 4981 | ZW | *Brassica oleracea* var. *botrytis* | RBOO00000000 | Dillon *et al*., 2019 |
| *P. syringae* pv. maculicola 90 32 | US, 1990 | *Brassica oleracea* | LGLH00000000 | N.A. |
| *P. syringae* pv. maculicola H7608 |  | *Brassica rapa* subsp*. oleifera* | LGLG00000000 | N.A. |
| *P. syringae* pv. maculicola ICMP 11281 | CN | *Brassica rapa* | RBUQ00000000 | Dillon *et al*., 2019 |
| *P. syringae* pv. maculicola ICMP 2744 | UK | *Brassica nigra* | RBQA00000000 | Dillon *et al*., 2019 |
| *P. syringae* pv. maculicola ICMP 3935 | NZ | *Brassica oleracea* var. *botrytis* | LJQR00000000 | Thakur *et al*., 2016 |
| *P. syringae* pv. maculicola M4a | US, 1965 | *Raphanus sativus* | LGLE00000000 | N.A. |
| *P. syringae* pv. persicae NCPPB 2254 | FR, 1969 | *Prunus persica* | ODAM00000000 | Ruinelli *et al*., 2019 |
| *P. syringae* pv. ribicola ICMP 3883 |  | *Ribes aureum* | RBNR00000000 | Dillon *et al*., 2019 |
| *P. syringae* pv. spinaceae ICMP 16928 | JP | *Spinacia oleracea* | RBTF00000000 | Dillon *et al*., 2019 |
| *P. syringae* pv. syringae B728a | US, 1987 | *Phaseolus vulgaris* | CP000075 | Feil *et al*., 2005 |
| *P. syringae* pv. syringae PD2766 | US | *Actinidia deliciosa* | LKEM00000000 | N.A. |
| *P. syringae* pv. tagetis ICMP 4092 | UK | *Tagetes erecta* | RBQC00000000 | Dillon *et al*., 2019 |
| *P. syringae* pv. theae ICMP 3923 | JP, 1970 | *Camellia sinensis* | LJRU00000000 | Thakur *et al*., 2016 |
| *P. syringae* pv. tomato K40 | USA, 2005 | *Solanum lycopersicum* | SAMN0000000 | N.A. |
| *P. syringae* pv. tomato Max13 | FR | *Solanum lycopersicum* | ADFZ00000000 | N.A. |
| *P. syringae* pv. tomato NCPPB1108 | UK, 1961 | *Solanum lycopersicum* | ADGA00000000 | N.A. |
| *P. syringae* pv. tomato T1 | 1986 | *Solanum lycopersicum* | ABSM00000000 | Almeida *et al*., 2009 |
| *P. syringae* pv. tomato 407 | US. 2004 | *Solanum lycopersicum* | LNKZ00000000 | Thapa and Coaker, 2016 |
| *P. syringae* pv. tomato A9 | US. 2005 | *Solanum lycopersicum* | LNKY00000000 | Thapa and Coaker, 2016 |
| *P. syringae* pv. tomato BRIP38746 | AU, 1973 | *Solanum lycopersicum* | SNVG00000000 | Griffin *et al.*, 2019 |
| *P. syringae* pv. tomato BRIP66796 | AU, 2015 | *Solanum lycopersicum* | SNVF00000000 | Griffin *et al.*, 2020 |
| *P. syringae* pv. tomato BRIP66810 | AU, 2015 | *Solanum lycopersicum* | SNVE00000000 | Griffin *et al.*, 2021 |
| *P. syringae* pv. tomato ICMP 2841 | DK | *Solanum lycopersicum* | RBUK00000000 | Dillon *et al*., 2019 |
| *P. syringae* pv. tomato ICMP 2844 | UK, 1960 | *Solanum lycopersicum* | LJRN00000000 | Thakur *et al*., 2016 |
| *P. syringae* pv. tomato ICMP 4263 | NZ | *Solanum lycopersicum* | RBRJ00000000 | N.A. |
| *P. syringae* pv. tomato ICMP 7230 | NZ | *Solanum lycopersicum* | RBRI00000000 | N.A. |
| *P. syringae* pv. tomato NYS-T1 | US, 2009 | *Solanum lycopersicum* | JRRA00000000 | Jones *et al.*, 2015 |
| *P. syringae* pv. tomato PT23 |  | *Solanum lycopersicum* | MSDS00000000 | Meaden and Koskella, 2017 |
| *P. syringae* pv. tomato delta IVIX |  | *Solanum lycopersicum* | CP047072 | N.A. |
| *P. syringae* pv. tomato delta VI |  | *Solanum lycopersicum* | CP047071 | N.A. |
| *P. syringae* pv. tomato delta X |  | *Solanum lycopersicum* | CP047073 | N.A. |
| *P. syringae* A7386 |  | *Cucumis sativus* | RBOB00000000 | Dillon *et al*., 2019 |
| *P. syringae* BS2900 |  |  | FNPP00000000 | N.A. |
| *P. syringae* PDD 32b 74 | FR, 2005 | air | MTSA00000000 | Besaury *et al*., 2017 |

**Table S2.** Genomic islands and prophages identified in *Pseudomonas syringae* pv. tomato DAPP-PG 215. Classification of identified prophages into intact (score > 90), questionable (score 70-90), and incomplete (score < 70) is done by PHASTER based on the presence of phage-like genes, size, and similarity to known phages.

| **Name** | **Location** | **Start (bp)** | **End (bp)** | **Size (bp)** | **GC %** | **CDS** | **Note** |
| --- | --- | --- | --- | --- | --- | --- | --- |
| GI-1 | Chromosome | 134144 | 139563 | 5419 | 52.85 | 7 |  |
| GI-2 | Chromosome | 228917 | 233808 | 4891 | 48.04 | 6 |  |
| GI-3 | Chromosome | 237698 | 249518 | 11820 | 54.02 | 16 |  |
| GI-4 | Chromosome | 252270 | 258286 | 6016 | 52.98 | 10 | *eriC, crcB* |
| GI-5 | Chromosome | 267958 | 270788 | 2830 | 52.98 | 5 |  |
| GI-6 | Chromosome | 275358 | 279146 | 3788 | 55.1 | 5 | *arsC, arsN1* |
| GI-7 | Chromosome | 474111 | 481728 | 7617 | 54.27 | 7 |  |
| GI-8 | Chromosome | 803316 | 805525 | 2209 | 53.81 | 4 |  |
| GI-9 | Chromosome | 808252 | 811575 | 3323 | 53.19 | 8 |  |
| GI-10 | Chromosome | 825986 | 829982 | 3996 | 51.96 | 4 | *hopD1, hopQ1-1* |
| GI-11 | Chromosome | 864351 | 867627 | 3276 | 47.27 | 3 | *avrA* |
| GI-12 | Chromosome | 882720 | 894919 | 12199 | 53.8 | 16 |  |
| GI-13 | Chromosome | 899039 | 906516 | 7477 | 50.37 | 10 | *hop-T1* |
| GI-14 | Chromosome | 907473 | 913151 | 5678 | 46.9 | 9 |  |
| GI-15 | Chromosome | 1124514 | 1127439 | 2925 | 47.37 | 3 |  |
| GI-16 | Chromosome | 1464536 | 1472851 | 8315 | 51.81 | 11 | *hopF2* |
| GI-17 | Chromosome | 1506800 | 1511810 | 5010 | 51.15 | 10 |  |
| GI-18 | Chromosome | 1736511 | 1740081 | 3570 | 54.43 | 6 |  |
| GI-19 | Chromosome | 1741976 | 1748489 | 6513 | 53.88 | 8 |  |
| GI-20 | Chromosome | 1771702 | 1778060 | 6358 | 54.53 | 8 |  |
| GI-21 | Chromosome | 1782695 | 1787242 | 4547 | 49.75 | 6 |  |
| GI-22 | Chromosome | 1818355 | 1833738 | 15383 | 48.15 | 5 |  |
| GI-23 | Chromosome | 1818355 | 1833738 | 15383 | 53.9 | 19 | *hopC1* |
| GI-24 | Chromosome | 1960161 | 1963133 | 2972 | 51.57 | 5 |  |
| GI-25 | Chromosome | 1965185 | 1973037 | 7852 | 47.62 | 7 |  |
| GI-26 | Chromosome | 2791500 | 2793770 | 2270 | 52.66 | 8 |  |
| GI-27 | Chromosome | 2866203 | 2870908 | 4705 | 48.13 | 8 |  |
| GI-28 | Chromosome | 2967638 | 2974386 | 6748 | 55.02 | 9 |  |
| GI-29 | Chromosome | 3880645 | 3885730 | 5085 | 46.48 | 5 |  |
| GI-30 | Chromosome | 3973090 | 3976931 | 3841 | 56.43 | 9 |  |
| GI-31 | Chromosome | 4397511 | 4403680 | 6169 | 51.04 | 5 |  |
| GI-32 | Chromosome | 4410176 | 4414526 | 4350 | 49.66 | 9 | *hopAF1* |
| GI-33 | Chromosome | 4495231 | 4499538 | 4307 | 52.84 | 6 |  |
| GI-34 | Chromosome | 4650506 | 4653339 | 2833 | 46.64 | 6 |  |
| GI-35 | Chromosome | 5074853 | 5080257 | 5404 | 53.37 | 8 | *hopT1-2, hopS* |
| GI-36 | Chromosome | 5136274 | 5138958 | 2684 | 52.24 | 5 |  |
| GI-37 | Chromosome | 5150450 | 5153919 | 3469 | 49.9 | 5 |  |
| GI-38 | Chromosome | 5465197 | 5467466 | 2269 | 52.06 | 4 |  |
| GI-39 | Chromosome | 5494967 | 5500153 | 5186 | 47.69 | 7 |  |
| GI-40 | Chromosome | 5504041 | 5509625 | 5584 | 54.02 | 9 |  |
| GI-41 | Chromosome | 5804073 | 5815124 | 11051 | 51.1 | 10 |  |
| GI-42 | Chromosome | 5850075 | 5853387 | 3312 | 46.12 | 6 |  |
| GI-43 | Chromosome | 5890460 | 5897338 | 6878 | 51.17 | 6 |  |
| GI-44 | Chromosome | 5958882 | 5964005 | 5123 | 50.32 | 10 |  |
| GI-45 | Plasmid p107 | 75700 | 70283 | 5417 | 49.26 | 9 |  |
| GI-46 | Plasmid p81 | 9452 | 21061 | 11609 | 51.72 | 11 | *avrD*, *hopD1*, *hopQ1-1* |
| GI-47 | Plasmid p81 | 22086 | 27535 | 5449 | 51.7 | 9 |  |
| PR-1 | Chromosome | 481728 | 489809 | 8081 | 58.24 | 6 | incomplete |
| PR-2 | Chromosome | 525093 | 543650 | 18557 | 59.64 | 23 | intact |
| PR-3 | Chromosome | 1111403 | 1119541 | 8138 | 53.89 | 8 | incomplete |
| PR-4 | Chromosome | 1463760 | 1489507 | 25747 | 55.54 | 13 | incomplete |
| PR-5 | Chromosome | 1480861 | 1505863 | 25002 | 59.74 | 30 | intact |
| PR-6 | Chromosome | 1728315 | 1739514 | 11199 | 56.27 | 10 | incomplete |
| PR-7 | Chromosome | 3563961 | 3622352 | 58391 | 58.58 | 64 | intact |
| PR-8 | Chromosome | 3871504 | 3884578 | 13074 | 51.52 | 14 | incomplete |
| PR-9 | Chromosome | 3912386 | 3977686 | 65300 | 57.74 | 94 | intact, *avrPto1* |
| PR-10 | Chromosome | 4340000 | 4346660 | 6660 | 58.73 | 7 | incomplete |
| PR-11 | Chromosome | 5806461 | 5852818 | 46357 | 56.36 | 49 | intact |
| PR-12 | Chromosome | 5852865 | 5887730 | 34865 | 58.59 | 42 | questionable |
| PR-13 | Plasmid p107 | 15075 | 21697 | 6622 | 54.46 | 7 | questionable |
| PR-14 | Plasmid p107 | 54234 | 65603 | 11369 | 55.07 | 16 | incomplete |

# References

Almeida, N. F., Yan, S., Lindeberg, M., Studholme, D. J., Schneider, D. J., Condon, B., Liu, H., Viana, C. J., Warren, A., Evans,C., Kemen, E., MacLean, D., Angot, A., Martin, G. B., Jones, J. D., Collmer, A., Setubal, C. J., and Vinatzer, B. A. (2009). A draft genome sequence of *Pseudomonas syringae* pv. tomato T1 reveals a type III effector repertoire significantly divergent from that of *Pseudomonas syringae* pv. tomato DC3000. *Mol. Plant-Microbe Interact. MPMI* 22, 52–62. doi: 10.1094/MPMI-22-1-0052.

Baltrus, D. A., Nishimura, M. T., Romanchuk, A., Chang, J. H., Mukhtar, Cherkis, K., Roach, J., Grant, S.R., Jones, C.D., and Dangl J. D. (2011). Dynamic evolution of pathogenicity revealed by sequencing and comparative genomics of 19 *Pseudomonas syringae* isolates. *PLoS Pathog.* 7, e1002132. doi: 10.1371/journal.ppat.1002132.

Baltrus, D. A., Yourstone, S., Lind, A., Guilbaud, C., Sands, D. C., Jones, C. D., Morris, C.E., Dangl, J. L. (2014). Draft genome sequences of a phylogenetically diverse Suite of *Pseudomonas syringae* strains from multiple source populations. *Genome Announc.* 2, e01195-13. doi: 10.1128/genomeA.01195-13.

Bartoli, C., Lamichhane, J. R., Berge, O., Guilbaud, C., Varvaro, L., Balestra, G. M., Vinatzer, B.A., and Morris, C.E. (2015). A framework to gauge the epidemic potential of plant pathogens in environmental reservoirs: the example of kiwifruit canker. *Mol. Plant Pathol.* 16, 137–149. doi: 10.1111/mpp.12167.

Besaury, L., Amato, P., Sancelme, M., and Delort, A. M. (2017). Draft genome sequence of *Pseudomonas syringae* PDD-32b-74, a model strain for ice-nucleation studies in the atmosphere. *Genome Announc.* 5, e00742-17. doi: 10.1128/genomeA.00742-17.

Buell, C. R., Joardar, V., Lindeberg, M., Selengut, J., Paulsen, I. T., Gwinn, M. L., Dodson, R. J., Deboy, R. T., Durkin, A. S., Kolonay, J. F., Madupu, R., Daugherty, S., Brinkac, L., Beanan, M. J., Haft, D. H., Nelson, W. C., Davidsen, T., Zafar, N., Zhou, L., Liu,J., Yuan, Q., Khouri, H., Fedorova, N., Tran, B., Russell, D., Berry, K., Utterback, T., Van Aken, S. E., Feldblyum, T. V., D'Ascenzo, M., Deng, W., Ramos A. R., Alfano, J. R., Cartinhour, S., Chatterjee, A. K., Delaney, T. P., Lazarowitz, S. G., Martin, G. B., Schneider, D. J., Tang, X., Bender, C. L., White, O., Fraser, C. M., and Collmer, A. (2003). The complete genome sequence of the *Arabidopsis* and tomato pathogen *Pseudomonas syringae* pv. tomato DC3000. *Proc. Natl. Acad. Sci. U. S. A.* 100, 10181–10186. doi: 10.1073/pnas.1731982100.

Buonaurio, R., Stravato, V. M., and Cappelli, C. (1996). Occurrence of *Pseudomonas syringae* pv. tomato race 1 in Italy on *Pto* gene-bearing tomato plants. *J. Phytopathol.* 144, 437–440. doi: 10.1111/j.1439-0434.1996.tb00320.x.

Dillon, M. M., Thakur, S., Almeida, R. N. D., Wang, P. W., Weir, B. S., and Guttman, D. S. (2019). Recombination of ecologically and evolutionarily significant loci maintains genetic cohesion in the *Pseudomonas syringae* species complex. *Genome Biol.* 20, 3. doi: 10.1186/s13059-018-1606-y.

Feil, H., Feil, W. S., Chain, P., Larimer, F., DiBartolo, G., Copeland, A., Lykidis, A., Trong, S., Nolan, M., Goltsman, E., Thiel, J., Malfatti, S., Loper, J. E., Lapidus, A., Detter, J. C., Land, M., Richardson, P. M., Kyrpides, N. C., Ivanova, N., and Lindow, S. E. (2005). Comparison of the complete genome sequences of *Pseudomonas syringae* pv. syringae B728a and pv. tomato DC3000. *Proc. Natl. Acad. Sci. U. S. A.* 102, 11064–11069. doi: 10.1073/pnas.0504930102.

Griffin, K., Campbell, P., and Gambley, C. (2019). Genetic basis of copper-tolerance in Australian *Pseudomonas syringae* pv. tomato. *Australas. Plant Pathol.* 48. doi: 10.1007/s13313-019-00646-y.

Jones, L. A., Saha, S., Collmer, A., Smart, C. D., and Lindeberg, M. (2015). Genome-assisted development of a diagnostic protocol for distinguishing high virulence *Pseudomonas syringae* pv. tomato strains. *Plant Dis.* 99, 527–534. doi: 10.1094/PDIS-08-14-0833-RE.

Kałużna, M., Willems, A., Pothier, J. F., Ruinelli, M., Sobiczewski, P., and Puławska, J. (2016). *Pseudomonas cerasi* sp. nov. (non Griffin, 1911) isolated from diseased tissue of cherry. *Syst. Appl. Microbiol.* 39, 370–377. doi: 10.1016/j.syapm.2016.05.005.

Mazo-Molina, C., Mainiero, S., Hind, S. R., Kraus, C. M., Vachev, M., Maviane-Macia, F., Lindeberg, M., Saha, S., Strickler, S. R., Feder, A., Giovannoni, J. J., Smart, C. D., Peeters, N., and Martin, G. B. (2019). The *Ptr1* locus of *Solanum lycopersicoides* confers resistance to race 1 strains of *Pseudomonas syringae* pv. tomato and to *Ralstonia pseudosolanacearum* by recognizing the type III effectors AvrRpt2 and RipBN. *Mol. Plant-Microbe Interact. MPMI* 32, 949–960. doi: 10.1094/MPMI-01-19-0018-R.

Meaden, S., and Koskella, B. (2017). Adaptation of the pathogen, *Pseudomonas syringae*, during experimental evolution on a native vs. alternative host plant. *Mol. Ecol.* 26, 1790–1801. doi: 10.1111/mec.14060.

Mott, G. A., Thakur, S., Smakowska, E., Wang, P. W., Belkhadir, Y., Desveaux, D., and Guttman, D. S. (2016). Genomic screens identify a new phytobacterial microbe-associated molecular pattern and the cognate Arabidopsis receptor-like kinase that mediates its immune elicitation. *Genome Biol*. 17, 98. doi: 10.1186/s13059-016-0955-7.

Ruinelli, M., Blom, J., Smits, T. H. M., and Pothier, J. F. (2022). Comparative genomics of *Prunus*-associated members of the *Pseudomonas syringae* species complex reveals traits supporting co-evolution and host adaptation. *Front. Microbiol.* 13, 804681. doi: 10.3389/fmicb.2022.804681.

Saitou, N., and Nei, M. (1987). The neighbor-joining method: a new method for reconstructing phylogenetic trees. *Mol. Biol. Evol.* 4, 406–425. doi: 10.1093/oxfordjournals.molbev.a040454.

Tamura, K., Stecher, G., and Kumar, S. (2021). MEGA11: Molecular evolutionary genetics analysis version 11. *Mol. Biol. Evol.* 38, 3022–3027. doi: 10.1093/molbev/msab120.

Thakur, S., Weir, B. S., and Guttman, D. S. (2016). Phytopathogen genome announcement: draft genome sequences of 62 *Pseudomonas syringae* type and pathotype strains. *Mol. Plant-Microbe Interact. MPMI* 29, 243–246. doi: 10.1094/MPMI-01-16-0013-TA.

Thapa, S. P., and Coaker, G. (2016). Genome sequences of two *Pseudomonas syringae* pv. tomato race 1 strains, isolated from tomato fields in California. *Genome Announc.* 4, e01671-15. doi: 10.1128/genomeA.01671-15.
